# Weed Hosts Represent an Important Reservoir of Turnip Yellows Virus and a Possible Source of Virus Introduction into Oilseed Rape Crop

**DOI:** 10.3390/v14112511

**Published:** 2022-11-13

**Authors:** Lucie Slavíková, Emad Ibrahim, Glenda Alquicer, Jana Tomašechová, Katarína Šoltys, Miroslav Glasa, Jiban Kumar Kundu

**Affiliations:** 1 Plant Virus and Vector Interactions, Crop Research Institute, Drnovská 507, Prague 16106, Czech Republic; 2 Department of Plant Protection, Faculty of Agrobiology, Food and Natural Resources, Czech University of Life Sciences Prague, Kamýcká 129, Prague 16500, Czech Republic; 3 Biomedical Research Center of the Slovak Academy of Sciences, Institute of Virology, Dúbravská Cesta 9, 84505 Bratislava, Slovakia; 4 Faculty of Natural Sciences, University of Ss. Cyril and Methodius, Nám. J. Herdu 2, 91701 Trnava, Slovakia; 5 Department of Microbiology and Virology, Comenius University in Bratislava, Ilkovičova 6, 84104 Bratislava, Slovakia

**Keywords:** TuYV, weed host, canola, RT-PCR, qPCR, HTS, RTD gene, natural reservoir

## Abstract

Turnip yellows virus (TuYV) is one of the most important pathogens of oilseed rape worldwide. The virus has a large host range including many crop species (e.g., oilseed rape, pea, chickpea) and weeds from more than twenty plant families. Other than oilseed rape, we detected TuYV in many commonly grown weed species that share the fields and vegetation period together with canola crops in Czech and Slovak Republics. TuYV was detected by reverse-transcription polymerase chain reaction (RT-PCR) in at least 26 species including main crop hosts (oilseed rape), intercrops and weeds such as *Amaranthus retroflexus*, *Atriplex patula (Amaranthaceae)*, *Arctium lappa*, *Lactuca serriola*, *Taraxacum officinale*, *Tripleurospermum inodorum (Asteraceae)*, *Phacelia tanacetifolia (Boraginaceae)*, *Brassica napus*, *Capsella bursa–pastoris*, *Descurainia Sophia*, *Raphanus raphanistrum*, *Sinapis alba*, *Sisymbrium officinale*, *Thlaspi arvense (Brassicaceae)*, *Silene alba*, *Stellaria media (Caryophyllaceae)*, *Euphorbia helioscopia (Euphorbiaceae)*, *Geranium rotundifolium (Geraniaceae)*, *Lamium purpureum (Lamiaceae)*, *Fumaria officinalis*, *Papaver rhoeas (Papaveraceae)*, *Veronica persica (Plantaginaceae syn. Scrophulariaceae)*, *Fallopia convolvulus (Polygonaceae)*, *Solanum nigrum (Solanaceae)*, *Urtica dioica (Urticaceae)* and *Viola arvensis (Violaceae)*. The detection of TuYV was further confirmed by RT-qPCR as well as Sanger sequencing of the PCR fragments. We discovered four new weed species as hosts of TuYV such as *T. inodorum*, *S. alba*, *G. rotundifolium* and *E. helioscopia*, representing their three respective plant families. The readthrough domain (RTD) gene sequence analysis of the Czech and Slovak TuYV isolates from oilseed rape and weed species showed similar within-group nucleotide divergence (7.1% and 5.6%, respectively) and the absence of geographical- or host-based phylogenetic clustering. The high-throughput sequencing of the *P. rhoeas* sample enabled the obtention of a nearly complete genome of TuYV and revealed the mixed infection of TuYV with turnip mosaic virus and cucumber mosaic virus. Our results thus show that weed species are an important TuYV reservoir and play a significant role in the spread and incidence of the disease in field crops such as oilseed rape.

## 1. Introduction

Oilseed rape (*Brassica napus*) is an important crop worldwide and has a long tradition in the European cropping system. Viral diseases are among the limiting biotic factors in oilseed rape cultivation. Four viral pathogens such as turnip yellows virus (TuYV, *Polerovirus*), turnip mosaic virus (TuMV, *Potyvirus*), broccoli necrotic yellows virus (BNYV, *Cytorhabdovirus*) and cauliflower mosaic virus (CaMV, *Caulimovirus*) are reported to be the main viruses infecting oilseed rape [1,2]. Among them, TuYV is the most important and causes significant losses in oilseed grain production. The high incidence of TuYV in oilseed rape crops could also have important consequences as it serves as a virus source for vegetable crops from *Brassicaceae* and other host families [3]. Yield reduction in plants showing severe virus symptoms due to TuYV has been estimated at 70 to 79% [3]. TuYV is transmitted by aphids in a persistent manner. The spread of the virus depends on the abundance and movement of the major aphid vectors, namely *Myzus persicae* and *Brevicoryne brassicae* [1], as well as virus reservoirs in weeds and volunteer host plants. TuYV has a broad host range, which includes, other than oilseed, a number of weeds [4] as well as economically important vegetable crops and intercrops [5]. Weed species serve not only as virus reservoirs but also as hosts for aphid vectors [6] and promote the spread of the virus to infect new plants and fields [7]. The weeds or cultivated hosts are also of concern for virus epidemiology because they overwinter and thus can constitute inoculum sources for early spring disease outbreaks in oilseed and vegetable crops [8,9,10]. Similarly, in many other cropping systems, weeds are the main reservoir of viruses infecting the field [2,11,12,13] and horticultural crops [14,15,16], especially insect-transmitted viruses [17,18].

TuYV belongs to the genus *Polerovirus* within the family *Solemoviridae* [19]. The virus was formerly known as beet western yellows virus (BWYV). TuYV has a single-stranded, positive-sense, approximately 5.6–6 kb RNA genome coding for at last six open reading frames (ORFs) [20]. ORFs 0, 1, and 2 are expressed from genomic RNA and ORFs 3, 4, and 5 are expressed from a subgenomic RNA. ORF 0 encodes a P0 protein that determines disease symptoms and host and acts as a silencing suppressor [21]. The P0 protein interacts with the RNA-induced silencing complex (RISC) and renders the complex inefficient by targeting the degradation of individual Argonaute 1 proteins before the RISC is formed [22]. ORF 1 and ORF 2 overlap and encode a replication-related protein that is individually expressed as a P1–2 fusion protein or a P1 protein [23]. The P2 protein encodes an RNA-dependent RNA polymerase (RdRp) and replicates viral genomic RNA using VPg as a primer template [24]. The major coat protein (CP, P3) is encoded by ORF 3, which is followed in the scaffold by ORF 5 (P5). P3–5 are expressed as a readthrough domain (RTD) protein and are required for aphid transmission and the efficient accumulation and spread of the virus in the plant [25,26,27]. An RTD is also the product of ORF 4 (P4), which is encoded within ORF 3 and is required for viral movement. A small ORF (P3a) is located in an intergenic region between ORF 2 and ORF 3. A viral genomic protein (VPg) is located at the 5′ end of the TuYV genome, as in all poleroviruses, and a 200-nucleotide-long non-coding region is located at the 3′ end [28].

A recent study has shown that TuYV isolates have a large diversity of genome sequences that contain few recombination events. The greatest molecular variability occurs in the RTD gene [10,29], which might be related to the different efficacy of the virus isolates in the aphids’ transmission [26,30].

In this work, we studied TuYV in oilseed rape crops, several weed species, and volunteer plants from different plant families (see Supplementary Table S1). These species are among the most important weeds in oilseed rape crops [31,32] and were present in large numbers in fields throughout the year, forming a “green belt” for the virus and vector. We detected TuYV in oilseed rape, 2 intercrop species and 23 weed species including 4 newly reported hosts such as *T. inodorum*, *S. alba*, *G. rotundifolium* and *E. helioscopia.* To study the genetic diversity of the virus in the Czech and Slovak republic, sequence analysis of selected isolates targeting the RTD domain of TuYV genome was performed. Furthermore, high-throughput sequencing (HTS) analysis of *P. rhoeas* with a multiple viral infection enabled us to determine the nearly complete genome of the TuYV isolate.

## 2. Materials and Methods

### 2.1. Sampling of Oilseed Rape and Weed Plants from Fields

Sampling was carried out randomly in oilseed rape fields in the vicinity of the Czech and Slovak Republic (Supplementary Table S1). The samples included *Brassica napus* (oilseed rape), *Sinapis alba, Phacelia tanacetifolia* (intercrop) and 38 weed species (including the most common for oilseed rape) from 17 plant families.

### 2.2. Detection of TuYV by RT-PCR

Plant samples were ground in liquid nitrogen, and RNA was extracted from 100 mg of leaf tissue. RNA was isolated using a Trizol kit (Thermo Scientific, Wilmington, DE, USA), according to the manufacturer’s instructions. The concentration and purity of the isolated RNA were then measured spectrophotometrically (NanoDrop 2000; Thermo Scientific, Wilmington, DE, USA). cDNA was prepared using 1.0 μg/μL of total RNA, a random hexamer primer and RevertAid RT (Thermo Scientific, Wilmington, DE, USA), according to the manufacturer’s instructions. The cDNA was amplified with the generic primer pair Luteovirus F/R as well as with the specific primers TuYV_5cpF/TuYVR-K2. The primers are listed in Supplementary Table S2 [33,34]. The 25 μL reaction mixture contained 1 μL cDNA, 1 μL each of 10 μmol forward and reverse primer, 12.5 μL 2× DreamTaq Green PCR Master Mix (Thermo Scientific, Wilmington, DE, USA) and 9.5 μL sterile nuclease-free water. PCR conditions were as follows: initial denaturation at 94 °C for 5 min, followed by 30 amplification cycles (94 °C, 45 s; 60 °C, 30 s; and 72 °C, 1 min), and a final extension at 72 °C for 10 min. The PCR product was then separated on a 1% agarose gel and stained with SYBR^®^ Safe DNA Gel Stain (Invitrogen, Wilmington, DE, USA).

### 2.3. Analysis of TuYV Titre in Oilseed Rape and Weed Plants by RT-qPCR

Oilseed rape, intercrop and weed samples from different fields were analyzed for TuYV titres using RT-qPCR. RT-qPCR was performed using a LightCycler^®^ 480 (Roche, Basel, Switzerland) with SYBR Green I as described in [35] and Dráb et al. [36]. Absolute quantification of viral RNA copies was performed using cDNA samples with a LightCycler^®^ 480 Instrument II (Roche, Basel, Switzerland) in 384-well plates containing 12 μL of reaction solutions per well. The PCR mastermix in each well consisted of the primer pair mix (0.5 μL of TuYVF-K1 and TuYVR-K1 or TuYVF-K2 and TuYVR-K2 at a final concentration of 10 µM; the primers are listed in Supplementary Table S2), 6 μL of LightCycler^®^ 480 SYBR Green I Master (Roche, Basel, Switzerland), and 4.5 μL of sterile nuclease-free water. Finally, 1 μL cDNA was added to this mixture to obtain a final volume of 12 μL. The thermal cycling protocol was as follows: 42 °C for 30 min, 95 °C for 10 min, followed by 40 cycles at 95 °C for 5 s, 60 °C for 30 s and 20 s at 72 °C. The fluorescence signal was measured at the end of each extension step at 72 °C. After amplification, a melting curve analysis was performed with a temperature gradient from 60 to 97 °C to confirm that only the specific products were amplified. Finally, the samples were cooled to 40 °C for 10 s. The fit points method in the software was used to determine the threshold for the cycle (Ct). TuYV copies in each plant sample were calculated using a standard curve generated by inserting the purified PCR fragment of the target sequence of TuYV (isolate No28dobrovizCZ, maintained in Chinese cabbage in Crop Research Institute) into the vector pGEM-T Easy Vector (Promega, Madison, WI, USA) and cloned into *E. coli* DH5a. The selected colony with confirmed insertion was cultured and the plasmid DNA was subsequently purified (Plasmid plus Midi Kit, Qiagen, Hilden, Germany). The amount of DNA was quantified by spectrophotometry (NanoDrop 2000; Thermo Scientific). To calculate the TuYV titre in the tested samples, a ten-fold standard dilution curve was constructed using the plasmid DNA of a positive TuYV sample. The plasmid quantity in μg was converted to pmol using the average molecular weight of a ribonucleotide (340 Da) and the number of bases of the transcript (Nb). The following mathematical formula was applied: pmol of dsDNA = μg of dsDNA) × 10^6^ pg μg^–1^/660 pmol pg^−1^/Nb. Avogadro’s constant (6.023 × 10^23^ molecules mol^–1^) was used to estimate the number of transcripts [27]. Standards and samples were prepared in triplicate. The resulting Ct value of 35 cycles was set as the threshold for virus-positive samples; all samples with a value above 35 were classified as negative.

### 2.4. Sequence Analysis of Readthrough Domain (RTD) Gene Sequence

A genomic region encompassing the RTD from a group of 26 isolates of oilseed rape, intercrops and weeds was determined from four overlapping fragments amplified using RT-PCR and the primer pairs listed in Table S2. Amplification of cDNA of TuYV isolates was carried out using high-fidelity Phusion High-Fidelity DNA Polymerase (Thermo Scientific, Waltham, MA, USA). The reaction conditions were as follows: initial denaturation at 94 °C for 2 min; followed by 30 amplification cycles of 94 °C for 10 s, 58 °C for 20 s and 72 °C for 30 s; and a final extension at 72 °C for 10 min. The PCR amplicons were purified by using QIAquick PCR Purification Kit according to the manufacture’s instruction (Qiagen, Hilden, Germany). PCR amplicons were commercially sequenced by using forward and reverse primers (Eurofins, Val Fleuri, Luxembourg). Thereafter, the sequences were edited using the bioinformatics tools Reverse Complement (https://www.bioinformatics.org/sms/rev_comp.html; accessed on 1 January 2000) and CAP3 assembly (https://doua.prabi.fr/cgi-bin/run_cap3; accessed on 1 January 2022), and the resulting contigs of nucleotide sequences were analyzed by Blast (NCBI: https://www.ncbi.nlm.nih.gov/; accessed on 1 January 2021). The determined partial TuYV genome sequences were deposited in the GenBank database under accession numbers OP699021-OP699053 (listed in Supplementary Table S3).

### 2.5. High-Throughput Analysis of the PK2 P. rhoeas Sample

A common poppy (*P. rhoeas*) plant (labelled as PK2) found in a garden in Pezinok (western Slovakia) in 2017 and showing severe symptoms involving mosaics and leaf deformations was analyzed using HTS. Total RNAs from symptomatic leaves were extracted using the NucleoSpin RNA Plant kit (Macherey-Nagel, Duren, Germany) and ribosomal RNA was removed using the Ribo-Zero rRNA Removal Kit (Illumina, San Diego, CA, USA). The rRNA-depleted total RNAs were used for double-stranded cDNA synthesis using the SuperScript II (Thermo Fisher Scientific, Waltham, MA, USA) kit. The sample was further processed with the transposon-based chemistry library preparation kit (Nextera XT, Illumina, San Diego, CA, USA) and the cDNA library was sequenced on the Illumina MiSeq platform (Illumina, San Diego, CA, USA, 300-bp paired-end sequencing). High-quality trimmed reads were used for de novo assembly and contigs were blasted to the viral genomes database (ftp://ftp.ncbi.nih.gov/genomes/Viruses/all.fna.tar.gz; accessed on 1 January 2022) using CLC Genomics Workbench 7.5 and Geneious v.8.1.9. The nearly complete TuYV genome sequence reported in this paper (missing 11 and 103 nt at the non-coding 5′ and 3′ extremities as compared to the reference NC_003743 genome) was deposited in the GenBank database under accession number OP562900.

### 2.6. Multiple Sequence Alignment and Phylogenetic Analysis

Multiple sequence alignment of nucleotide and amino acid sequences was per-formed using Clustal X (version 2.0) [37] with default parameters, and phylogenetic trees were generated using Clustal X and MEGA 7 [38] (www.megasoftware.net/; accessed on 1 January 2022) applying maximum likelihood method. The reliability of branches was inferred from bootstrap analysis of 1000 replicates. The analysis of TuYV diversity was performed using DnaSP software [39].

## 3. Results and Discussion

### 3.1. Detection of TuYV in Oilseed Rape and Weed Hosts

**Figure 1:**
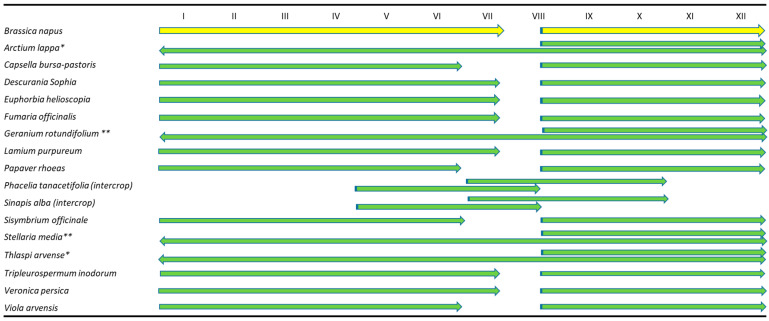
The growing season of oilseed rape and weeds covers the whole year. Most species are annual weeds, with the exception of *A. lappa, T. arvense* (* either annual or bi-annual) and *S. media*, *G. rotundifolium* (** either annual or perennial). (The blue color indicates the beginning of the growing season).

**Figure 2:**
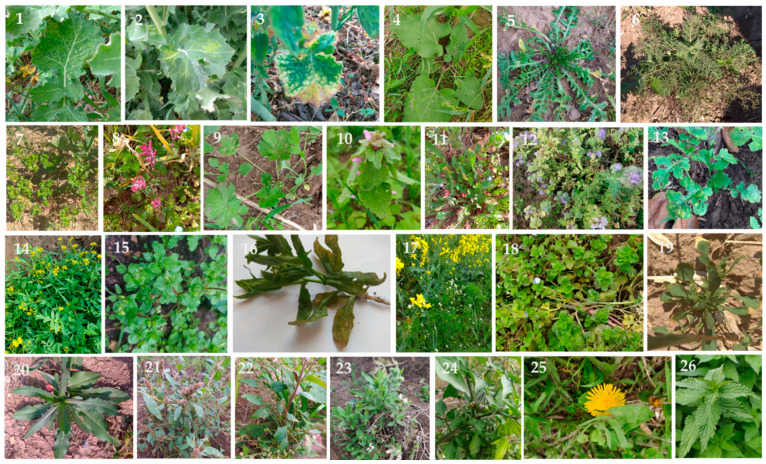
Oilseed rape, intercrops and weed species infected by TuYV. (**1**–**3**): Brassica napus, (**4**): Arctium lappa, (**5**): Capsella bursa-pastoris, (**6**): Descurainia sophia, (**7**): Euphorbia helioscopia, (**8**): Fumaria officinalis, (**9**): Geranium rotundifolium, (**10**): Lamium purpureum, (**11**): Papaver rhoeas, (**12**): Phacelia tanacetifolia (intercrop), (**13**): Sinapis alba (intercrop), (**14**): Sisymbrium officinale, (**15**): Stellaria media, (**16**): Thlaspi arvense, (**17**): Tripleurospermum inodoorum, (**18**): Veronica persica, (**19**): Viola arvensis, (**20**): Lactuca seriola, (**21**): Amaranthus retroflexus, (**22**): Atriplex patula, (**23**): Silene alba, (**24**): Solanum nigrum, (**25**): Taraxacum officinalis, (**26**): Urtica dioica.

We detected TuYV in 160 out of 417 samples tested, using RT-PCR from different species from different plant families, including *Amaranthaceae: Amaranthus retroflexus, Atriplex patula; Asteraceae: Arctium lappa, Lactuca serriola, Taraxacum officinale, Tripleurospermum inodorum; Boraginaceae: Phacelia tanacetifolia; Brassicaceae: Brassica napus, Capsella bursa–pastoris, Descurainia Sophia, Raphanus raphanistrum, Sinapis alba, Sisysimbrium officinale, Thlaspi arvense; Caryophyllaceae: Silene alba, Stellaria media; Euphorbiaceae: Euphorbia helioscopia; Geraniaceae: Geranium rotundifolium; Lamiaceae: Lamium purpureum; Papaveraceae: Fumaria officinalis, Papaver rhoeas; Plantaginaceae (Scrophulariaceae): Veronica persica; Polygonaceae: Fallopia convolvulus; Solanaceae: Solanum nigrum; Urticaceae: Urtica dioica;* and *Violaceae: Viola arvensis* (Table S1, Figure 1 and Figure 2). As previously shown, the main hosts of TuYV in arable crops belong to families such as *Brassicaceae, Fabaceae, Chenopodiaceae* and *Asteraceae* [5,8,9,40]. Moreover, many weed species from different plant families serve as hosts for the virus, namely *Amaranthaceae, Apiaceae, Asteraceae, Boraginaceae, Brassicacae, Caryophyllaceae, Cucurbitaceae, Euphorbiaceae, Fabaceae, Fumarianceae, Geraniaceae, Hydrophyllaceae, Malvaceae, Oxalidaceae, Lamiaceae, Papaveraceae, Polemoniaceae, Portulaceae, Primulaceae, Rhizophoraceae, Scrophulariaceae, Solanacecae, Tropaeolaceae, Urticaceae,* and *Violaceae* [2,5,8,9,28,40,41,42]. However, most of these host data date back from the earlier reports related to the host range of BWYV. Recent data on the newly classified TuYV linked to the weed hosts or intercrop species are still limited. Our results confirm that in addition to oilseed rape and two intercrops (*P. tanacetifolia* and *S. alba*), at least 23 weed species serve as hosts for TuYV, including four new host species from *Asteraceae (T. inodorum), Caryophyllaceae (S. alba)*, *Geraniaceae* (*G. rotundifolium),* and *Euphorbiaceae (E. helioscopia)* families. The host range of TuYV thus appears to be very wide among weed species, as it so far includes plant species from 24 families that are commonly grown in and within annual crops worldwide, such as brassicas, fabacious and sugar beet plants. The weed species in which TuYV has been detected in the Czech and Slovak Republics are either winter species that share a common growing season with oilseed rape (Figure 1 and Figure 2) or spring weeds or volunteer plants, which makes them a very effective reservoir for the virus and aphid vectors. The winter species such as *V. arvensis, T. arvense, C. bursa-pastoris, D. sophia F. officinalis* and *G. rotundifolium* are the most important weeds for oilseed rape [30,31,43]. The spices such as *S. nigrum, A. retroflexus* and *A. patula* are usually spring weeds of broad-row crops (e.g., sugar beet, cruciferous and other species of vegetables) [44] and apparently represent TuYV sources from spring to summer, transmitting the virus to winter weed species as well as crops, mainly by cabbage aphids (*Brevicoryne brassicae*). The green peach aphids (*Myzus persicae*) afterward transmit the virus to newly emerging winter crops in early autumn [2,9,45]. Species such as *A. lappa, S. alba,* and *U. dioica* mainly grow on the field margins as volunteer plants [31] and are additional reservoirs. In addition, biennial weeds such as *A. lappa, V. persica* (both annual or biennials) and some perennial spices of *Conyza spp.* [9] or *G. rotundifolium* can be a permanent reservoir for TuYV and provide an overwintering green-bridge for the disease cycle. Important reservoir hosts for TuYV are also the species used for intercropping green manures, such as *S. alba* or *P. tanacetifolia,* which usually cover the green gap between the post-harvest period and the newly emerging oilseed rape in the fields (Figure 1). The broad and diverse host range of TuYV that includes cultivated, volunteer and weed plants as well as plants grown in winter, spring, and summer, ensures that the virus is constantly maintained in the ecosystem and can be easily transmitted to field crops by aphids [8,46]. Apart from the fact that weeds often grow near or within fields and orchards, the cultivated varieties of some weeds can be used as ornamental plants. Moreover, weed seeds can lie dormant in the soil for a long period and are easy to grow, thus they are abundant in the environment [47] and are important components of disease control in crops. 

### 3.2. TuYV Titre in Oilseed Rape and Weed Plants 

**Figure 3:**
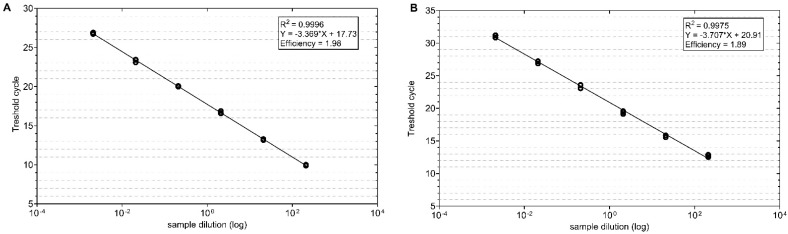
Standard curves for SYBR Green-I-based real-time RT-PCR amplification of standard TuYV DNA with specific primer sets (**A**) TuYV K1 and (**B**) TuYV K2. The amplification plots show the testing, in triplicate, of a ten-fold dilution series containing standard TuYV DNA from 2.1 × 10^2^ to 2.1 × 10^4^ template copies/reaction.

RT-qPCR was performed using two primer pairs TuYVF-K1/TuYVR-K1 and TuYVF-K2 and TuYVR-K2. The Ct values were measured in triplicate and plotted against the known copy numbers of the standard sample. The standard curve covered a linear range of five orders of magnitude. For the TuYV K1 primers, the slope was −3.369, intersection = 17.73, r^2^ = 0.9996 and efficiency = 1.98; for the TuYV K2 primers, the slope was −3.707, intersection = 20.91, r^2^ = 0.9975 and efficiency = 1.89 (Figure 3A,B) (2.0 being a theoretical perfect efficiency, indicating the doubling of product with every cycle). The laboratory isolate of TuYV was used for the qPCR optimization and both primers in the RT-qPCR assay successfully amplified their respective targets (see Supplementary Table S2). The copies of transcripts are equivalent to the viral gene copies. 

**Figure 4:**
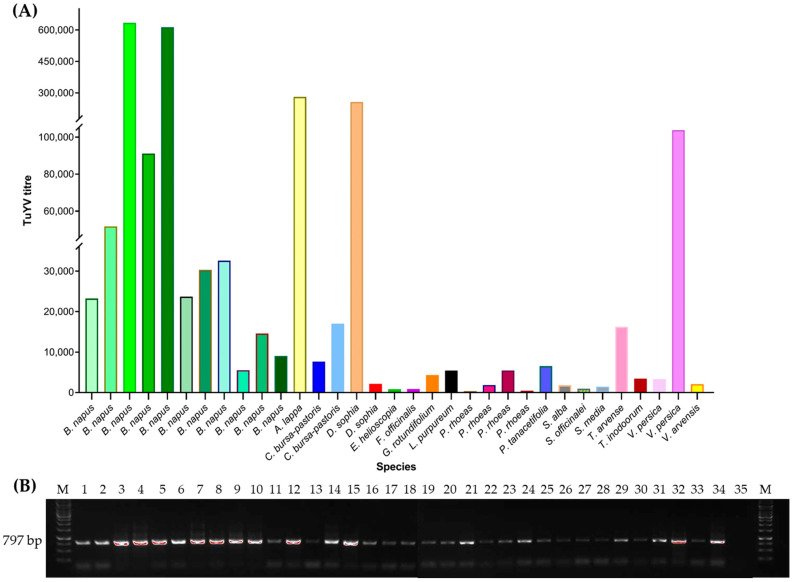
(**A**). TuYV titre detected in RT-PCR-positive plants of oilseed rape, intercrop and weed species by RT-qPCR. (**B**). TuYV detection by RT-PCR using the primer pair TuYV_5cpF/TuYVR-K2, 797 bp (lanes 1–33, representing the same samples as in Figure **A** {from left to right}; lanes 34 and 35 positive and negative control, respectively; lane M GeneRuler 1 kb DNA ladder, Thermo fisher Scientific, Wilmington, DE, USA).

The RT-qPCR assay using the primer pair TuYVF-K1/TuYVR-K1 was used to detect the viral titre to confirm the RT-PCR-based detection in the field samples. The detection of the virus titre by RT-qPCR ranged from a few hundred to thousands of virus copies. In most of the weeds, the virus titre remained low, with the exception of *A. lappa, D. sophia* and *V. persica*, where titres similar to *B. napus* were detected (Figure 4).

### 3.3. The Diversity of TuYV Sequences from Crop and Non-Crop Host

**Figure 5:**
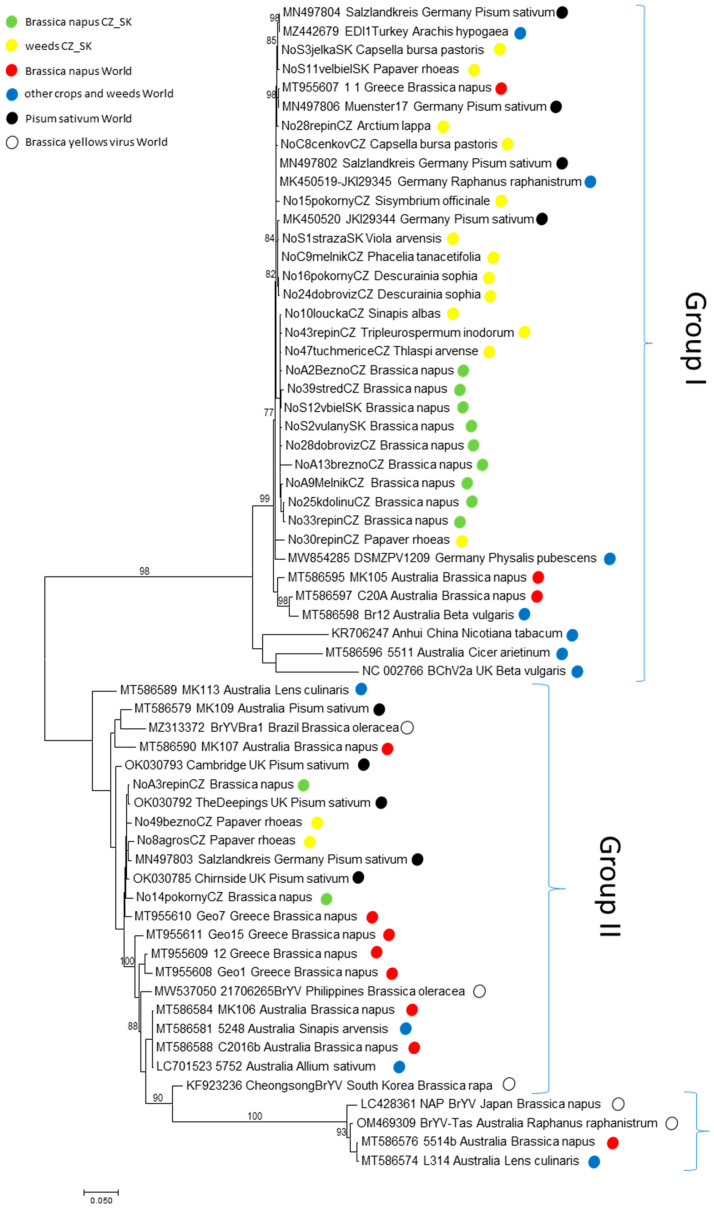
Maximum likelihood phylogenetic tree constructed from alignments of partial TuYV sequences of the genomic regions encompassing the RTD domain (nts 3483–5508 based on the reference NC_003743 genome). The Czech, Slovak and selected Genbank sequences of previously characterized isolates are identified by their accession number, isolate name and geographical location. Only bootstrap values ≥ 70% (1000 bootstrap resamplings) are indicated.

To assess the genetic diversity of TuYV in the Czech and Slovak regions, we analyzed 26 TuYV isolates in a ca. 2040 bp-long genome portion encoding the P3–P5 readthrough domain (RTD), which is required for virus circulation, accumulation and persistence in the aphid vector [24,25,26]. The phylogenetic analysis revealed that TuYV isolates, including those available in Genbank, were split into three major groups (Figure 5). Most of the Czech and Slovak clustered in Group I, irrespective of their host origin. Only two Czech isolates from *B. napus* and two isolates from *P. rhoeas* were assigned to Group II, while no isolate was found belonging to Group III. Based on the phylogenetic analyses, no clear-cut clustering was noted among TuYV isolates in the examined genomic region. The calculation of the within mean group nucleotide distance (p-distance model) between Czech and Slovak *B. napus* (*n* = 11) and weed isolates (*n* = 15) showed similar values of 7.1% and 5.6%, respectively. The similar diversity within both populations and the same between-group distance (6.2%) may indicate the absence of particular selection pressure imposed on “culture” and “wild” TuYV isolates and the presumed mixing of both populations during the vegetation period due to vector transmission.

### 3.4. Virome Analysis of a Common Poppy (P. rhoeas) Plant 

**Figure 6:**
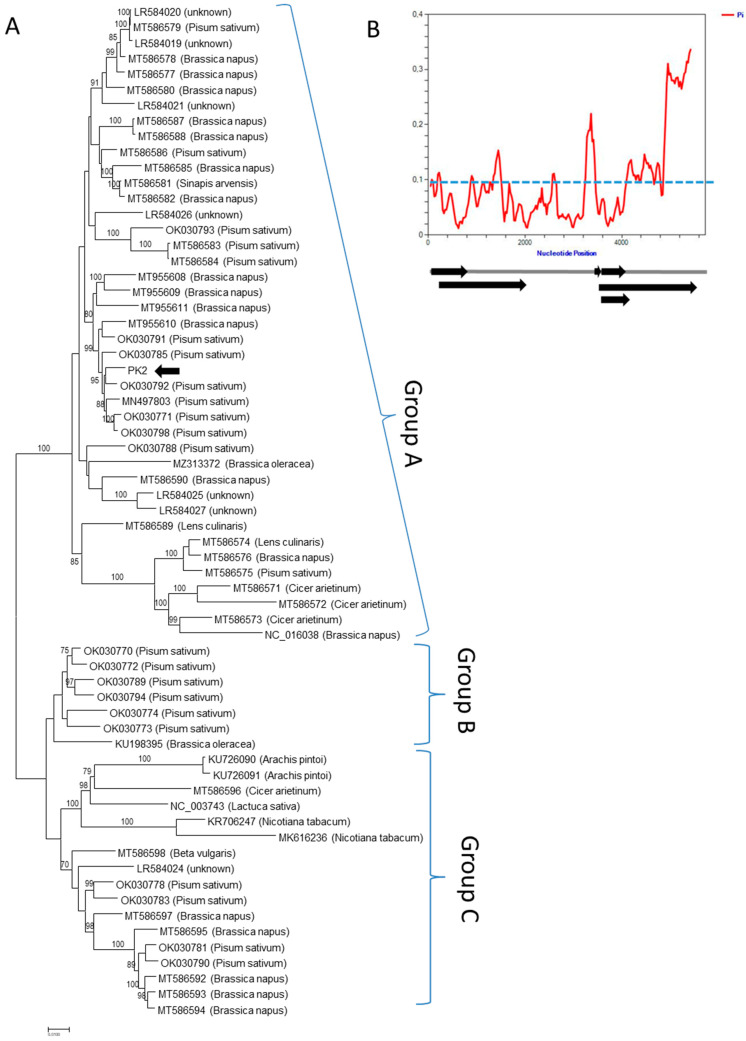
(**A**). Phylogenetic tree generated on available nearly complete nucleotide genome sequences of TuYV isolates (*n* = 65). The phylogenetic analysis was inferred using maximum likelihood (ML) based on the General Time Reversible (GTR) model selected as the best-fit model of nucleotide substitution based on Bayesian information criterion (BIC) as implemented in MEGA X. Isolates are identified by their GenBank accession numbers and host (if known). The Slovak PK2 isolate sequenced in the present study is highlighted by an arrow. The scale bar indicates a genetic distance of 0.01. Bootstrap values higher than 70% (1000 bootstrap resamplings) are indicated. (**B**). Nucleotide diversity (π) plotted along the TuYV genomes with a window of 100 nt and a step of 25 nt. The blue dotted line indicates the mean nucleotide diversity (π = 9.2%) within TuYV genomes. In scale schematic representation of TuYV genome is shown below.

In an effort to further compare the genetic relatedness of a “weed” TuYV isolate to known TuYV diversity, the nearly complete genome of the PK2 isolate from additional common poppy (*P. rhoeas*) was determined from HTS data (available in Genbank under accession number OP562900). The HTS approach provides an unbiased access to the plant virome and viral diversity studies, overcoming the limits of conventional detection tools [48]. The analysis of the generated PK2 sequence data (ca. 3.9 millions of reads with a mean length of 144.8 nts) enabled us to obtain a large contig of ca. 5.3 kb, corresponding to TuYV. Subsequent rounds of mapping of individual reads against the TuYV genomes allowed the validation and reconstruction of the nearly complete genome (5477 nts, 677 reads mapped, with a mean coverage of 17.1×). Interestingly, the phylogenetic analysis placed this isolate in the same cluster as *Pisum sativum* isolates from the UK and Germany (Figure 6), further confirming the absence of host-related phylogenetic groups within TuYV. Similarly, the BLAST searches identified the *P. sativum* isolates OK030792, MN497803 and OK030785 as the closest PK2 relatives with more than 98% nt identity. Interestingly, RTD- (Figure 5) and nearly complete genome-based phylogenetic trees (Figure 6) display slightly different topology, probably due to recombination event(s) in the TuYV evolutionary history affecting different genome portions [28]. HTS revealed that besides TuYV, the PK2 plant was co-infected by two additional viruses, i.e., cucumber mosaic virus and turnip mosaic virus. Consequently, due to the mixed infection, the potential role of individual viruses in the observed symptomatology (Supplementary Figure S1) could not be determined. Complex viral infection of plants is a common phenomenon [49], possibly further enhancing the viral disease impact and complicating the phytosanitary management efforts. 

## 4. Conclusions

We detected TuYV in oilseed rape fields in both crops and in various weeds, including winter and spring species as well as volunteer plants. These species are important annual weeds both winter and spring (or some species can be bi-annual e.g., *A. lappa, T. inodorum,* and perennial, e.g., *S. media, G. rotundifolium*) in central Europe. Some weed species share a common growing season with oilseed rape, and others are spring weeds and therefore serve as reservoirs for the virus and the aphid vectors (e.g., *Brevicoryne brassicae, Myzus persicae*) during both pre- and post-harvest periods throughout the vegetation. Similarly, intercrop species (*S. alba* or *P. tanacetifolia*) could play a particularly important role in the disease cycle of TuYV as they harbor both the virus and the aphid vectors and close the green gap between crops. A molecular analysis of the RTD coding region showed that there is no strict regional or host-specific clustering of TuYV isolates, suggesting a long-term diversification of the virus, easy gene flow between crop and wild plant communities, and inter-regional spread of the virus through infected plant material. As the virome analysis of the *P. rhoeas* plant shows, the infection of weeds can be complex and involve different viruses. Due to the reliance on insecticidal seed treatments, the occurrence of the virus becomes a threat to oilseeds or other host plants (e.g., *Brassica spps.*, *Pisum vulgaris*, *Cicer arietinum Lupinus albus Vicia faba*, *Lens culinaris*, *Spinacea oleracea*, *Lactuca sativa*). Therefore, weed control as well as the control of aphids in intercrops can significantly reduce disease pressure and the incidence of TuYV in field crops, including oilseed rape.

## Data Availability

All data used in this study are publicly available on NCBI. A list of the accession numbers used is found in Supplementary Table S3.

## Supplementary Materials

The following supporting information can be downloaded at: https://www.mdpi.com/article/10.3390/v14112511/s1, Table S1: The plant sample tested for turnip yellows virus; Table S2. Primers used in this study; Table S3: TuYV isolates from the Czech and Slovak Republics and reference isolates of the readthrough domain (RTD) gene sequence analyzed in this study; Figure S1: Common poppy PK2 plant naturally mixed-infected with TuYV, CMV and TuMV.
