# Supplementary material for: Weed Hosts Represent an Important Reservoir of Turnip Yellows Virus and a Possible Source of Virus Introduction into Oilseed Rape Crop
Source: Viruses. 2022 Nov 13;14(11):2511. doi: 10.3390/v14112511 (PMC9696028; doi:10.3390/v14112511)
Supplement: Supplementary file 1 [file viruses-14-02511-s001.zip › viruses-1999651-supplementary/Supplementary Files/Supplementary Table S1-S2-S3.pdf]

viruses MDPI

Supplementary Table S1. The plant sample tested for turnip yellows virus.

Plant Species	Plant Family	Number of Tested Plant	Number of TuYV Detected Plant by RT-PCR
Achillea millefolium	*Asteraceae*	1	0
Amaranthus retroflexus	*Amaranthaceae*	1	1
Anchusa arvensis	*Boraginaceae*	2	0
Arctium lappa	*Asteraceae*	2	1
Atriplex patula	*Amaranthaceae*	2	2
Barbarea vulgaris	*Brassicaceae*	2	0
Brassica napus	*Brassicaceae*	184	78
Brassica napus (volunteer)	*Brassicaceae*	15	7
Capsella bursa - pastoris	*Brassicaceae*	10	2
Descurainia sophia	*Brassicaceae*	14	8
Euphorbia helioscopia	*Euphorbiaceae*	10	3
Fagopyrum esculentum	*Polygonaceae*	2	0
Fallopia convolvulus	*Polygonaceae*	2	1
Fumaria officinalis	*Papaveraceae*	13	4
Galinsoga parviflora	*Asteraceae*	1	0
Galium aparine	*Rubiaceae*	2	0
Geranium rotundifolium	*Geraniaceae*	6	2
Chenopodium album	*Amaranthaceae*	4	0
Lactuca serriola	*Asteraceae*	3	1
Lamium purpureum	*Lamiaceae*	4	1
Lotus corniculatus	*Fabaceae*	1	0
Mercurialis annua	*Euphorbiaceae*	6	0
Papaver rhoeas	*Papaveraceae*	12	6
Phacelia tanacetifolia (intercrop)	*Boraginaceae*	4	3
Plantago lanceolata	*Plantaginaceae*	3	0
Raphanus raphanistrum	*Brassicaceae*	5	3
Rumex obtusifolius	*Polygonaceae*	2	0
Silene alba	*Caryophyllaceae*	7	1
Sinapis alba (intercrop)	*Brassicaceae*	25	14
Solanum nigrum	*Solanaceae*	1	1
Sonchus arvensis	*Asteraceae*	3	0
Stellaria media	*Caryophyllaceae*	4	1
Sisysimbrium officinale	*Brassicaceae*	2	1
Taraxacum officinale	*Asteraceae*	5	1
Thlaspi arvense	*Brassicaceae*	13	6
Trifolium repens	*Fabaceae*	2	0

Tripleurospermum inodorum	*Asteraceae*	3	1
Urtica dioica	*Urticaceae*	5	1
Veronica persica	*Plantaginaceae*	4	1
Vicia hirsuta	*Fabaceae*	2	0
Viola arvensis	*Violaceae*	24	9
**Total**	**17**	**413**	**160**

Supplementary Table S2. Primers used in this study.

Primer Name	Sequence (3´-5´)	Nucleotide Position (MN497806)	PCR Fragment Length (bp)	Detection Methods	qPCR Efficiency	Reference
luteoviruses-F	GCTCTAGAATTGTTAATGAITACGGTCG	Generic Primery	610	RT-PCR		[33]
luteoviruses-R	CACGCGTCIACCTATTTIGGITTITG					
TuYVF-K1	TACGGTCGTGGGTAGGAGAA	3488-3507	185	qPCR	1.98	In this study
TuYVR-K1	TCGAACCTGCTCCTCTGGTA	3672-3653				
TuYVF-K2	GAGCTGGACCCACACTGTA	3867-3885	136		1.90	[34]
TuYVR-K2	ATTGGTCCTCGGCAACGTCG	4002-3983				
TuYV_5cpF	ATCCAGCGTCCGTTGAACTT	3206-3225	797	RT-PCR/Sequencing		In this study
TuYVR-K2	ATTGGTCCTCGGCAACGTCG	4002-3983				[34]
TuYV_5rtpF	CGACCTAGACGACGACGAAG	3603-3622	1072	Sequencing		In this study
TuYV_5rtpR	CAGCGGGTCCAACAACAAAG	4674-4655				
TuYV_3rtpF	AACCAACCTCAAGCACCACA	4399-4418	670			
TuYVF6-AS	TTTGACGACATAGCCTCTTC	5068-5049				
TuYV3terF1	CCGGAGAAGGAAAACTCCGA	4857-4876	696			
TuYV3terR1	TCGCCTCGTATCACTACCAAC	5552-5532				

Table S3. TuYV isolates from the Czech and Slovak Republics and reference isolates of the read-through domain (RTD) gene sequence analyzed in this study.

**Host**	**Location**	**Virus Isolate**	**Accession Number**
Brassica napus	Praha, Czech Republic	No28dobrovizCZ	OP699023
Brassica napus	Praha, Czech Republic	No39stredCZ	OP699024
Brassica napus	Kladno, Czech Republic	No14pokornyCZ	OP699025
Brassica napus	Kladno, Czech Republic	No25kdolinuCZ	OP699026
Brassica napus	Mělník, Czech Republic	No33repinCZ	OP699027
Brassica napus	Mělník, Czech Republic	NoA3repinCZ	OP699028
Brassica napus	Mělník, Czech Republic	NoA9melnikCZ	OP699029
Brassica napus	Mladé Boleslav, Czech Republic	NoA13breznoCZ	OP699030
Brassica napus	Chomutov, Czech Republic	NoA2beznoCZ	OP699031
Brassica napus	Senec, Slovak Republic	NoS12vbielSK	OP699032
Brassica napus	Galanta, Slovak Republic	NoS2vulanySK	OP699033
Arctium lappa	Mělník, Czech Republic	No28repinCZ	OP699034
Capsella bursa-pastoris	Praha, Czech Republic	NoC8cenkovCZ	OP699035
Capsella bursa-pastoris	Galanta, Slovak Republic	NoS3jelkaSK	OP699036
Descurainia sophia	Kladno, Czech Republic	No16pokornyCZ	OP699037
Descurainia sophia	Praha, Czech Republic	No24dobrovizCZ	OP699038
Euphorbia helioscopia#	Mělník, Czech Republic	No35repinCZ	OP699049
Fumaria officinalis#	Mělník, Czech Republic	No29repinCZ	OP699050
Geranium rotundifolium#	Kladno, Czech Republic	No12pokornyCZ	OP699021
Lamium purpureum#	Mělník, Czech Republic	No37repinCZ	OP699022
Papaver rhoeas	Kladno, Czech Republic	No8agrosCZ	OP699039
Papaver rhoeas	Mělník, Czech Republic	No30repinCZ	OP699040
Papaver rhoeas	Chomutov, Czech Republic	No49beznoCZ	OP699041
Papaver rhoeas	Senec, Slovak Republic	NoS11velbielSK	OP699042
Phacelia tanacetifolia	Mělník, Czech Republic	NoC9melnikCZ	OP699043
Sinapis alba	Kladno, Czech Republic	No10louckaCZ	OP699044
Sisymbrium officinale	Kladno, Czech Republic	No15pokornyCZ	OP699045
Stellaria media#	Kladno, Czech Republic	No3zajezdCZ	OP699051
Thlaspi arvense	Praha, Czech Republic	No47tuchmericeCZ	OP699046
Tripleurospermum inodoorum	Mělník, Czech Republic	No43repinCZ	OP699047
Veronica persica#	Kladno, Czech Republic	No3agrosCZ	OP699053
Veronica persica#	Galanta, Slovak Republic	No4jelkaSK	OP699052
Viola arvensis	Žlina, Slovak Republic	NoS1strazaSK	OP699048
Brassica napus	Greece	Geo15	MT955611
Brassica napus	Greece	Geo7	MT955610
Brassica napus	Greece	1_1	MT955607

Brassica napus	Greece	12	MT955609
Brassica napus	Greece	Geo1	MT955608
Brassica napus	Australia	MK106	MT586584
Brassica napus	Australia	C2016b	MT586588
Brassica napus	Australia	MK105	MT586595
Brassica napus	Australia	C20	MT586597
Brassica napus	Australia	5514b	MT586576
Brassica napus	Australia	MK107	MT586590
Brassica napus	Australia	5514b	MT586576
Brassica napus	Japan	NAPBrYV	LC428361
Lactuca sativa	France	FL1	NC_003743
Pisum sativum	UK	The Deepings	OK030792
Pisum sativum	UK	Cambridge	OK030793
Pisum sativum	UK	Canterbury	OK030767
Pisum sativum	UK	Chirnside	OK030785
Pisum sativum	Germany	Salzlandkreis	MN497803
Pisum sativum	Germany	Muenster17	MN497806
Pisum sativum	Germany	JKI29344	MK450520
Pisum sativum	Germany	Salzlandkreis	MN497802
Pisum sativum	Germany	Salzlandkreis	MN497804
Pisum sativum	Australia	MK109	MT586579
Sinapis arvensis	Australia	5248	MT586581
Lens culinaris	Australia	MK113	MT586589
Allium sativum	Australia	5752	LC701523
Lens culinaris	Australia	L314	MT586574
Arachis hypogaea	Turkey	EDI1	MZ442679
Raphanus raphanistrum	Germany	JKI29345	MK450519
Physalis pubescens	Germany	DSMZPV1209	MW854285
Beta vulgaris	Australia	Br12	MT586598
Cicer arietinum	Australia	5511	MT586596
Nicotiana tabacum	China	Anhui	KR706247
Brassica oleracea	Philippines	21706265BrYV	MW537050
Brassica oleracea	Brazil	BrYV Bra1	MZ313372
Brassica rapa	South_Korea	CheongsongBrYV	KF923236
Raphanus raphanistrum	Australia	BrYV-Tas	OM469309
Beta vulgaris	UK	BChV2a	NC_002766

#*partial* RTD sequence and not included in sequence analysis.
