# Supplementary figures and images for: Weed Hosts Represent an Important Reservoir of Turnip Yellows Virus and a Possible Source of Virus Introduction into Oilseed Rape Crop
Source: Viruses. 2022 Nov 13;14(11):2511. doi: 10.3390/v14112511 (PMC9696028; doi:10.3390/v14112511)

## Slide 1
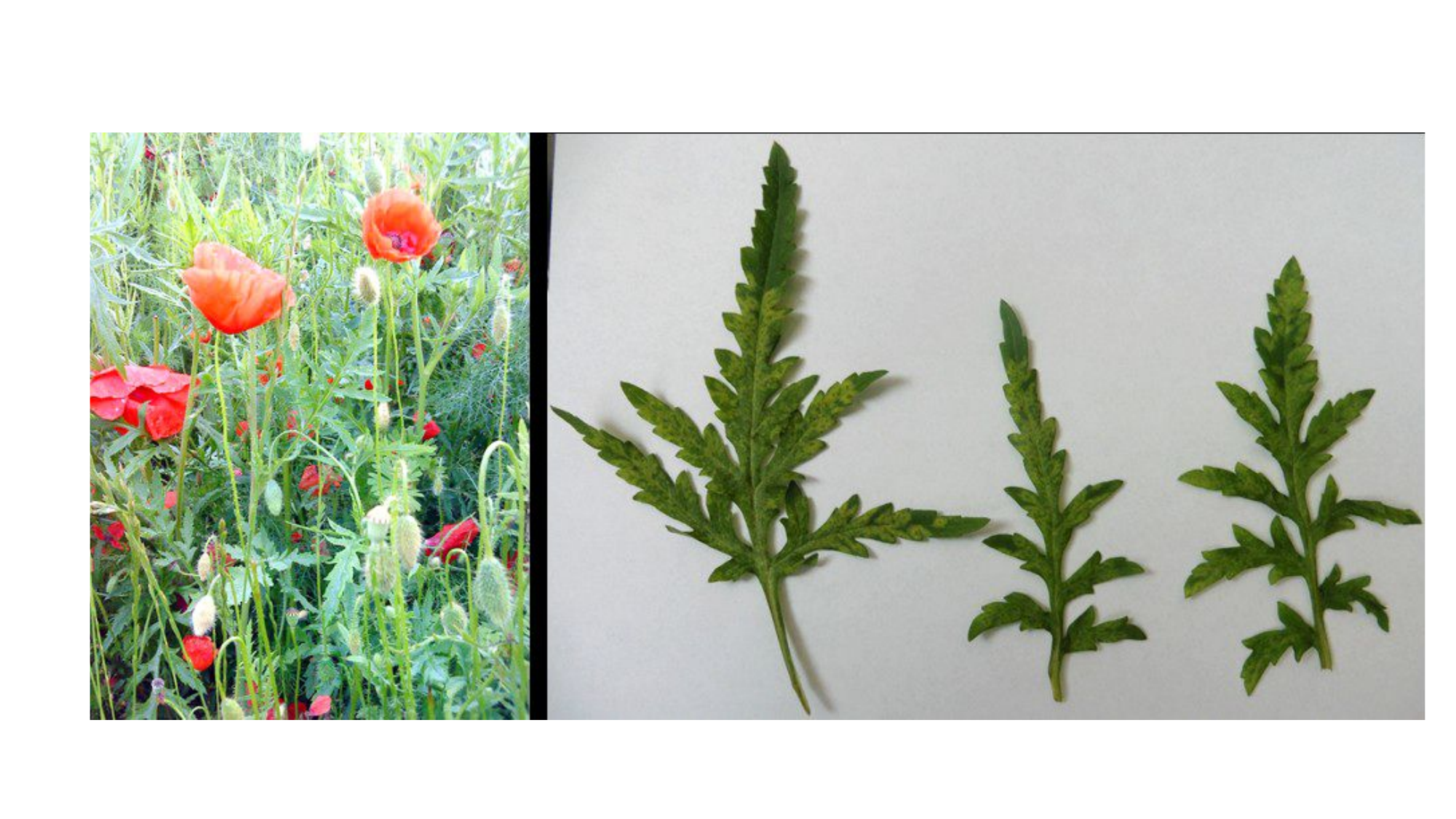

Supplement: Supplementary file 1 [file viruses-14-02511-s001.zip › viruses-1999651-supplementary/Supplementary Files/Supplementary Figure S1.pptx]
